# Editorial: Interventions to prevent or reduce unhealthy habits in children and adolescents during restricted conditions

**DOI:** 10.3389/fpubh.2024.1517367

**Published:** 2024-12-10

**Authors:** Mojtaba Keikha, Mohammad Hossein Ebrahimi, Mostafa Dianati-Nasab, Motahar Heidari-Beni

**Affiliations:** ^1^Department of Biostatistics and Epidemiology, Faculty of Public Health, Kerman University of Medical Sciences, Kerman, Iran; ^2^Environmental and Occupational Health Research Center, Shahroud University of Medical Sciences, Shahroud, Iran; ^3^School of Medical and Life Sciences, Sunway University, Sunway City, Malaysia; ^4^Department of Complex Genetics and Epidemiology, School of Nutrition and Translational Research in Metabolism, Maastricht University, Maastricht, Netherlands; ^5^Department of Nutrition, Child Growth and Development Research Center, Research Institute for Primordial Prevention of Non-Communicable Disease, Isfahan University of Medical Sciences, Isfahan, Iran

**Keywords:** child and adolescence, healthy behavior, intervention, lock down, COVID-19, obesity, screen time

The prevalence of childhood overweight and obesity has increased worldwide in recent decades. Childhood obesity is associated with serious health problems and the risk of premature illness and death later in life (1, 2). Obesity is linked to cardiometabolic risk factors such as insulin resistance, type 2 diabetes, hyperlipidemia, and hypertension, all of which can lead to cardiometabolic disease. Several important risk factors such as low physical activity, sedentary behavior, increased screen time activity and high fast-food intake lead to overweight and obesity in children and adolescents (3, 4).

In a situation such as the COVID-19 pandemic, due to physical distancing, school or university closures and other preventive precautions, all the potential mentioned risk factors were more probable. Many studies during the COVID-19 pandemic show that children were more overweight, had lower levels of physical activity, and spent more time on screens (5, 6).

In the foreseeable future, the world may encounter a situation such as the COVID-19 pandemic again, and society should be ready for a clear response. Since many healthy and unhealthy habits are formed during childhood and adolescence, and many of these habits are irreversible, the current Research Topic entitled “*Interventions to prevent or reduce unhealthy habits in children and adolescents during restricted conditions*” was developed to propose new and novel strategies to modify vulnerable conditions. In the first article in this Research Topic, Kuzik et al., sought to update a comprehensive national assessment of physical activity and related behaviors, characteristics, and opportunities for children and youth (ParticipACTION Report Card on Physical Activity for Children and Youth) during the COVID-19 pandemic. The research team captured the best data on physical activity throughout the COVID-19 period, which was synthesized across 14 different indicators in four categories. The authors conclude that during the COVID-19 pandemic, the overall physical activity score decreased from a D+ (2020) to a D, coinciding with a decline in scores reflecting fewer opportunities for sports and community/facility-based activities and higher levels of sedentary behaviors. Fortunately, improvements in Active Transportation and Active Play during COVID-19 prevented a worse shift in children's health behaviors.

Li et al., assessed the mediating role of self-esteem in the relationship between family functioning and problematic Internet pornography use (PIPU). Their results showed that self-esteem partially mediated the relationship between family functioning and PIPU. The authors concluded that for adolescents with high belongingness needs who are at high risk for PIPU, good family functioning may have a protective effect by boosting self-esteem.

Barsch et al., tried to answer the question of whether sports boarding schools pose a particular risk of infection for students. They found that in a single-center prospective cohort study, no significant group difference between sports boarding schools and day schools was detected with respect to the number of COVID-19 infections. Their results indicate that sports boarding schools do not pose an increased risk of infection, assuming that the facilities prevent viral transmission with appropriate preventive strategies and hygiene measures.

Lee B. et al., examined the relationship between the playfulness experienced by middle school students during their early morning exercise and their physical self-efficacy and education for happiness. The authors reported that middle school playfulness had a significant effect on physical self-efficacy and a significant effect on education for happiness. In another study, Major et al., examined changes in screen time and its components (screen time spent on videos, games, homework, and other activities) of adolescents affected by COVID-19 school closures compared to controls from pre-pandemic years. They reported that COVID-19-related school closures modified and increased age-specific increases in screen time for both boys and girls. In a study exploring the relationship between perceived school climate and exercise behavior among obese adolescents, Yin et al. observed that perceived school climate among obese adolescents positively predicted exercise behavior and that obese adolescents' perception of school climate can effectively enhance their motivation to participate in exercise behavior and indirectly influence exercise behavior through exercise benefits and perseverance qualities.

In another article, Irschik et al., assessed changes in BMI and weight development in children during and (in particular) after the COVID-19 restrictions in Austria. The researchers reported that the rate of obesity increased by 88.5%, from 6.4 to 12.1%, during the pandemic, reaching a maximum of 15.2% during the restrictions. With the exception of obese children, all children in the study population experienced significant weight loss after the restrictions were lifted. Obese children continued to gain weight without any sign of normalization. In another work, Saintila et al. tried to determine the association between social network addiction (SNA) and anxiety symptoms with the risk of metabolic syndrome (MetS) in adolescents. The authors concluded that SNA and the presence of anxiety symptoms are associated with MetS.

In a study conducted by Qi et al., the research team aimed to determine the mediating role of school-based rope-skipping sports participation (SRSP) in the connection between social support and moderate- to vigorous physical activity (MVPA). They surveyed 721 adolescents residing in Changsha City and found a significant influence of the interaction between increased participation in and social support on school children's engagement in MVPA. An open-label randomized control trial by Kaur et al., assessed the effectiveness of the program to lower unwanted media screen time (PLUMS) among children aged 2–5 years in Chandigarh, Union Territory, North India. They found that the PLUMS intervention significantly reduced children's mean ST on a typical day and increased physical activity immediately post-intervention and during the 6-month follow-up period.

In their study to assess the relationship between parental anxiety and adolescent internet addiction, Wang et al. showed that family environment and adolescent emotional behavior issues played an indirect role in the link between parental anxiety and internet addiction. Their findings emphasize the importance of addressing parental anxiety and fostering a positive family environment as effective measures to alleviate adolescent emotional behavior problems and reduce the risk of internet addiction. In a web-based survey by Yuan et al., the authors evaluated parental knowledge of myopia control and explored its change during the outbreak of the COVID-19 pandemic. They showed that the COVID-19 pandemic obviously changed children's daily routines. More efforts should be made to narrow the gap between knowledge and behavior of myopia control, and to stay alert to the potential increased risk of myopia after the COVID-19 pandemic.

In a study conducted by Pope et al. to identify community settings and intervention strategies to prioritize for an intervention promoting healthy weight in rural preschool children, the authors reported that priority intervention strategies included providing nutrition and physical activity education, increasing access to healthy foods and physical activity in the built environment, and enhancing food security and their findings will be useful for the development of a multi-level community-based intervention. Finally, the last article in this Research Topic was authored by Lee Y.-R. et al., to explore how early childhood teachers (ECTs) can improve their personal resilience to adapt to and cope with disasters as part of early childhood education and care. According to their findings general disaster preparedness must be improved, followed by the development of strategies to strengthen children's resilience and work-related disaster preparedness.

In conclusion, the articles included in this Research Topic point out the importance of preventing the risk factors for obesity in children and adolescents and also highlight the burden of obesity in some situations where limitations are in place, such as the COVID-19 pandemic of 2020. To prevent this issue and act in the future, we need planning and programs at the family, community, school or university level.
